# Mapping the Multi-Organ miRNA-mRNA Regulatory Network in LPS-Mediated Endotoxemic Mice: Exploring the Shared Underlying Key Genes and Mechanisms

**DOI:** 10.3389/fmolb.2020.573327

**Published:** 2020-11-24

**Authors:** Cong Zhang, Ying Liao, Zhihao Liu, Lijin Zeng, Zhihua Peng, Jinli Liao, Zhen Yang

**Affiliations:** ^1^Division of Emergency Medicine, Department of Emergency Intensive Care Unit, The First Affiliated Hospital, Sun Yat-sen University, Guangzhou, China; ^2^Department of Cardiology, The First Affiliated Hospital, Sun Yat-sen University, Guangzhou, China; ^3^NHC Key Laboratory on Assisted Circulation (Sun Yat-sen University), Guangzhou, China; ^4^Department of Medical, Dermatology Hospital, Southern Medical University, Guangzhou, China

**Keywords:** lipopolysaccharide (LPS), multi-organ damage, gene expression omnibus (GEO), functional enrichment analysis, key genes

## Abstract

**Background:**

To this day, the molecular mechanism of endotoxin-induced multi-organ failure has not been completely clarified. This study aimed to construct an miRNA-mRNA regulatory network and identify main pathways and key genes in multi-organ of LPS-mediated endotoxemic mice.

**Methods:**

Public datasets from six mRNA and three miRNA microarray datasets were downloaded from the GEO website to screen final differentially expressed genes (FDEGs) and hub genes in the heart, lung, liver, and kidney of LPS-mediated endotoxemic mice. Functional and pathway enrichment analysis of FDEGs was used to identify the main pathways in multi-organ damage of LPS-treated mice. Finally, hub genes of each organ were intersected to obtain the key genes of multi-organ.

**Results:**

Firstly, 158, 358, 299, and 91 FDEGs were identified in the heart, lung, liver, and kidney, respectively. The pathway enrichment analysis of the FDEGs then showed that the TNF signaling pathway, Toll-like receptor signaling pathway, and some viral-infection-related pathways (influenza A, measles, and herpes simplex) were the main pathways in multi-organ damage of LPS-mediated endotoxemic mice. Moreover, miRNA-mRNA or PPI regulatory networks were constructed based on FDEGs. According to these networks, 31, 34, 34, and 31 hub genes were identified in the heart, lung, liver, and kidney, respectively. Among them, nine key genes (Cd274, Cxcl1, Cxcl9, Icam1, Ifit2, Isg15, Stat1, Tlr2, and Usp18) were enriched in Toll-like receptor signaling pathway and chemokine signaling pathway. Finally, seven potential drugs were predicted based on these key genes.

**Conclusion:**

The shared underlying molecular pathways in endotoxin-induced multi-organ damage that have been identified include Toll-like receptor signaling pathway and TNF signaling pathway. Besides, nine key genes (Cd274, Cxcl1, Cxcl9, Icam1, Ifit2, Isg15, Stat1, Tlr2, and Usp18) and seven potential drugs were identified. Our data provide a new sight and potential target for future therapy in endotoxemia-induced multi-organ failure.

## Introduction

Endotoxemia is caused by the release of endotoxin from bacteria in the blood and the translocation of endotoxin from intestinal bacteria, which can further cause a systemic inflammatory response and lead to multiple organ failure (Wu et al., 1996; Goligorsky and Sun, 2020). There is no specific drug treatment for endotoxin-mediated multi-organ injury in the clinic, which may lead to high mortality. To this date, the molecular mechanism of endotoxin-induced multi-organ failure has not been completely clarified. Lipopolysaccharide (LPS), also known as endotoxin, makes up the main content of the cell wall of Gram-negative bacteria and plays an important role in the pathogenesis of endotoxemia (Schneider and Ayres, 2008; Fu and Blackshear, 2017). Previous studies have confirmed that LPS can cause organ damage through Toll-like receptor signaling, proinflammatory signaling, oxidative stress, and apoptosis pathways (Cochet and Peri, 2017; Deng et al., 2020; Gao et al., 2020; Wei et al., 2020). There may be a common mechanism in LPS-induced multi-organ damage, which may provide potential therapeutic targets for multi-organ failure. However, most of the previous studies only focused on the LPS-induced single organ damage. It is therefore urgent to study the gene or microRNA (miRNA) regulatory network and common mechanisms of multiple organs in LPS-induced multi-organ damage (LIMOD).

microRNA is a type of highly conserved endogenous non-coding RNA that can regulate gene expression by inducing mRNA degradation or translation inhibition at the post-transcriptional level (Brandenburger and Lorenzen, 2020). Previous study found that miRNAs were differentially expressed in LPS-induced organ injury, indicating that they could be acted as potential molecular markers for disease diagnosis and therapeutic targets (Talepoor et al., 2017; Song et al., 2020; Wei et al., 2020). Generally, miRNAs negatively regulate the expression of genes. Delineating the miRNA-mRNA regulatory network may therefore help us to find new therapeutic targets. In our study, we analyzed the public datasets and described the miRNA–mRNA network or protein–protein interaction network and screened the hub genes of the heart, lung, liver, and kidney in LPS-treated mice via bioinformatics methods. Besides, we identified the key genes by crossing the hub genes of all organs to find the common mechanism in all organs damaged by LPS. Moreover, we also predicted the possible therapeutic drugs of these genes via the drug–gene database, which may provide a new therapeutic direction for endotoxin-induced multi-organ failure in the future.

## Materials and Methods

### Study Workflow

**FIGURE 1:**
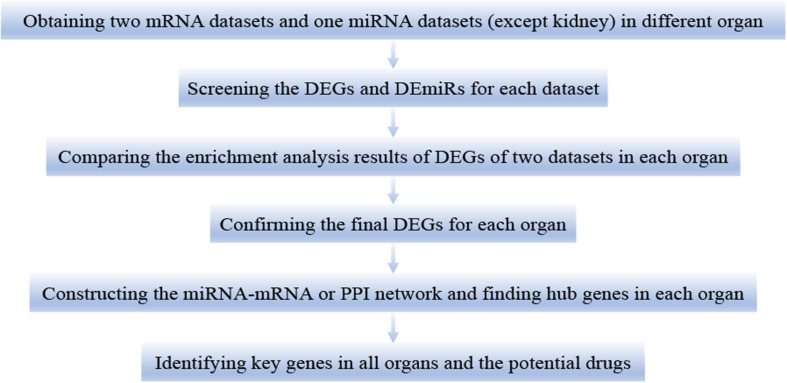
The workflow of this study. DEGs, differentially expressed genes. DEmiRs, differentially expressed miRNAs.

This study was divided into the following six steps (Figure 1). First, we downloaded two mRNA and one miRNA (except kidney) expression datasets of different organs (heart, lung, liver, and kidney) in LPS treated mice. Second, we calculated the differentially expressed genes (DEGs) and differentially expressed miRNAs (DEmiRs) of each dataset during LPS treatment. Third, we used the DEGs for functional enrichment analysis in each dataset. By comparing the results of enrichment analysis of two mRNA expression datasets of each organ, we confirmed that the two mRNA expression datasets of each organ were homogeneous. Fourth, we obtained the final DEGs (FDEGs) from the intersection of DEGs from the two datasets in each organ. Fifth, by using miRNA targets prediction software, we predicted the target genes of DEmiRs in each organ (except kidney). Besides, we get the possible target genes of DEmiRs in each organ by crossing the predicted target genes with the previously obtained FDEGs. Then we constructed miRNA-mRNA regulatory network or PPI network in each organ and screened hub genes in each organ. Finally, through the intersection of the hub genes of all organs, we identified the key genes in LPS induced multi-organ damage and predicted the possible therapeutic drugs through the drug database.

### Microarray Data Source

All microarray data were downloaded from the public database GEO database^1^. We choose different datasets in different organs, and each dataset was divided into the control and LPS group. In the heart, we chose GSE35934, GSE63920, and GSE29914 datasets (Drosatos et al., 2011; Li et al., 2012; Drosatos et al., 2016). Besides this, we selected the GSE59404, GSE130936, and GSE36472 datasets in the lung (Hsieh et al., 2012; Haspel et al., 2014; Petersen et al., 2019). Moreover, we selected GSE33901, GSE35934, and GSE33902 datasets in the liver (Li et al., 2012; Warg et al., 2012). Furthermore, we selected GSE35934 and GSE120879 datasets in the kidney (Li et al., 2012; Hato et al., 2019). The details for these datasets used in this study were shown in Table 1. It is noted that all datasets we used in this study could be downloaded on the website, and this part does not involve animal or human experiments by the authors.

**TABLE 1:** The detail information of used GSE datasets in this research.

Organ	GSE datasets	GSM used for analysis	Mouse strain	Age	Gender	Dose and time of LPS	Administration route
Heart	GSE35934 (mRNA)	CTR (GSM877596, GSM877597, GSM877598)	FVB	6–8 w	Female	4 mg/kg, 4 h	ip
		LPS (GSM877599, GSM877600, GSM877601)					
	GSE63920 (mRNA)	CTR (GSM1560369, GSM1560370, GSM1560371, GSM1560372)	C57BL/6	12 w	Female	5 mg/kg, 7–9 h	ip
		LPS (GSM1560365, GSM1560366, GSM1560367, GSM1560368)					
	GSE29914 (miRNA)	CTR (GSM740681, GSM740682, GSM740683, GSM740684)	C57BL/6	12 w	Female	5 mg/kg, 7–9 h	ip
		LPS (GSM740677, GSM740678, GSM740679, GSM740680)					
Lung	GSE59404 (mRNA)	CTR (GSM1436437, GSM1436471, GSM1436480)	C57BL/6J	4–6 w	Male	12 mg/kg, 8 h	ip
		LPS (GSM1436382, GSM1436386, GSM1436460)					
	GSE130936 (mRNA)	CTR (GSM3756516, GSM3756517, GSM3756518, GSM3756525)	C57BL/6J	–	Male	20 mg/kg, 7 h	iv
		LPS (GSM3756519, GSM3756520, GSM3756521)					
	GSE36472 (miRNA)	CTR (GSM894363, GSM894364, GSM894365)	C57BL/6	4–6 w	–	100 μg, 6 h	ip
		LPS (GSM894366, GSM894367, GSM894368)					
Liver	GSE33901 (mRNA)	CTR (GSM838585, GSM838586, GSM838587, GSM838588, GSM838589, GSM838590)	B6 × 129 F2 mice	6–8 w	Male	30 mg/kg, 6 h	ip
		LPS (GSM838597, GSM838598, GSM838599, GSM838600)					
	GSE35934 (mRNA)	CTR (GSM877677, GSM877678, GSM877679)	FVB	6–8 w	Female	4 mg/kg, 4 h	ip
		LPS (GSM877680, GSM877681, GSM877682)					
	GSE33902 (miRNA)	CTR (GSM838553, GSM838554, GSM838555, GSM838556, GSM838557, GSM838558)	B6 × 129 F2 mice	6–8w	Male	30 mg/kg, 6 h	ip
		CTR (GSM838565, GSM838566, GSM838567, GSM838568)					
Kidney	GSE35934 (mRNA)	CTR (GSM877605, GSM877606, GSM877607)	FVB	6–8 w	Female	4 mg/kg, 4 h	ip
		LPS (GSM877671, GSM877672, GSM877673)					
	GSE120879 (mRNA)	CTR (GSM3417707, GSM3417708, GSM3417709, GSM3417710)	C57BL/6J	8–12 w	Male	5 mg/kg, 4 h	iv
		LPS (GSM3417711, GSM3417712, GSM3417713, GSM3417714)					

*GSM, sample accessions numbers; CTR, control group; LPS, lipopolysaccharide; Dose and time of LPS, the dose of LPS used in LPS group and the time for obtaining specimen after LPS treatment; ip, intraperitoneal injection; iv, intravenous administration.*

### The Identification of DEGs and DEmiRs

After downloading the datasets, we used R (3.6.1 vision) to perform data quality control, remove missing values, perform data correction, and transfer the probe IDs to gene symbols. The limma package of R was used for screening DEGs or DEmiRs between control and LPS treated mice. Cutoff value (| logFC| > 1.5 and adjusted *p*-value <0.05) was used for screening DEGs or DEmiRs.

### The Functional Enrichment Analysis of DEGs or FDEGs

The function enrichment analysis was constructed by Gene Ontology (GO) and Kyoto Encyclopedia of Genes and Genomes (KEGG) pathways on Database for Annotation, Visualization, and Integrated Discovery (DAVID) website^2^ (Huang Da et al., 2009). GO term enrichment analysis mainly contains cellular component (CC), biological process (BP), and molecular function (MF), and we mainly chose the results of BP. The DEGs or FDEGs were used separately for enrichment analysis. The cutoff value was set as *p* < 0.05 and enrichment counts >2.

### The Prediction of Target Genes for DemiRs

miRecords online website^3^ was applied to search the predicted target genes of DEmiRNAs (Xiao et al., 2009). The inclusion standard of target gene prediction is that it can be predicted from more than 3 of 11 miRNA-gene prediction datasets (MicroInspector, Rusinov et al., 2005; miRanda, John et al., 2004; DIANA-microT, Kiriakidou et al., 2004; RNAhybrid, Kruger and Rehmsmeier, 2006; miTarget, Kim et al., 2006; MirTarget2, Wang and El Naqa, 2008; PicTar, Krek et al., 2005; PITA, Kertesz et al., 2007; RNA22, Miranda et al., 2006; NBmiRTar, Yousef et al., 2007; and Target Scan, Agarwal et al., 2015).

### Interaction and Regulatory Network Establishment of Selected FDEGs

The Search Tool for the Retrieval of Interacting Genes (STRING) database was used for the construction of the protein–protein interaction (PPI) network. The FDEGs of each organ were applied for this network, respectively. Moreover, the miRNA–mRNA network was based on the constructed PPI network or the network of DEmiRs with their predicted paired FDEGs. Finally, these relationship data were analyzed and mapping by Cytoscape software (version 3.7.2). The top 30 in the degree of nodes were selected as hub genes.

### The Key Genes and Drugs of Four Organs in LPS-Treated Mice

The key genes were identified by the intersection of the hub genes in all organs. We then used these key genes to screen the potential drugs in LPS induced multi-organ dysfunction by online DGIdb^4^ tool, which presents the drug–gene interaction based on the combination of expert curation and text mining (Cotto et al., 2018).

### Animal Experimental Protocol

Twelve male C57BL/6J mice (aged 6–8 weeks old, 18–20 g) were obtained from NBRI (Nanjing Biomedical Research Institute of Nanjing University, China). After the mice adapt to the environment, they were randomly divided into two groups: the control group and the LPS group. Each group included six mice. The control group and LPS group were intraperitoneally injected with PBS and LPS (Sigma, 10 mg/Kg), respectively (Qi et al., 2016; Zhai and Guo, 2016; Liu et al., 2017; Proniewski et al., 2019; Wepler et al., 2019). Twelve mice were euthanized, and the samples of the heart, lung, liver, and kidney were harvested at 6 h after LPS or PBS treatment. The study protocol was carried out according to the rules and regulations of Institutional Animal Care and Use Committee of Sun Yat-sen University. All experimental procedures in the *in vivo* mouse model were approved by the Institutional Animal Care and Use Committee of Sun Yat-sen University.

### Quantitative Real-Time Polymerase Chain Reaction

First, the total RNA of each organ (heart, lung, liver, and kidney) was obtained using Trizol reagent (Invitrogen). Then, RNA was reverse transcribed into cDNA using one High Capacity cDNA Reverse Transcription Kit (Roche) in accordance with the manufacturer’s instructions. Real-time polymerase chain reaction (RT-PCR) was used to detect the mRNA levels of Cd274, Cxcl1, Cxcl9, Icam1, Ifit2, Isg15, Stat1, Tlr2, and Usp18 in the heart, lung, liver, and kidney tissues by the SYBR Green (Takara) dye detection procedure. Gapdh was chosen as the reference gene. The primers were as follows: Cd274, 5′-TGTCACTTGCTACGGGCGTT-3′ (forward) and 5′-TCTCCCCCTGAAGTTGCTGT-3′ (reverse); Ifit2, 5′-GTTACACAGCAGTCATGAGTACAA-3′ (forward) and 5′-TGCAGTGCTTTACATAGGCCA-3′ (reverse); Isg15, 5′-GAC GCAGACTGTAGACACGC-3′ (forward) and 5′-CTGTGCACT GGGGCTTTAGG-3′ (reverse); Stat1, 5′-GCTGCCTATGATGT CTCGTTT-3′ (forward) and 5′-TGCTTTTCCGTATGTTGTG CT-3′ (reverse); Tlr2, 5′-TCAAGGAGGTGCGGACTGTT-3′ (forward) and 5′-GCATCCTCTGAGATTTGACGCTT-3′ (re- verse); Usp18, 5′-GATCACGGACACAGACTTGACAG-3′ (for- ward) and 5′-ACTCTTTGGGCTGGACGAAAC-3′ (reverse); Cxcl9, 5′-GAAAAGCCTTCCCTGGCAGA-3′ (forward) and 5′- GGCTCAAGGGCGTGATGAAA-3′ (reverse); Cxcl1, 5′-ACTGC ACCCAAACCGAAGTC-3′ (forward) and 5′-GTTACTTGGGG ACACCTTTTAGCA-3′ (reverse); Icam1, 5′-GCCAATTTCTCA TGCCGCAC-3′ (forward) and 5′-GCCTTCCAGGGAGCAAAA CA-3′ (reverse); and Gapdh, 5′-AGGTCGGTGTGAACGGATT TG-3′ (forward) and 5′-CAGTAGAGGCAGGGATGATGTTCT-3′ (reverse).

### Statistical Analysis

In this research, all data were analyzed by SPSS 25.0 software. The results of each group were presented as the mean ± SEM. Significance analysis was performed by Student’s *t*-test. *P* < 0.05 was set as statistically significant.

## Results

### The miRNA-mRNA Network of Heart in LPS-Treated Mice

**FIGURE 2:**
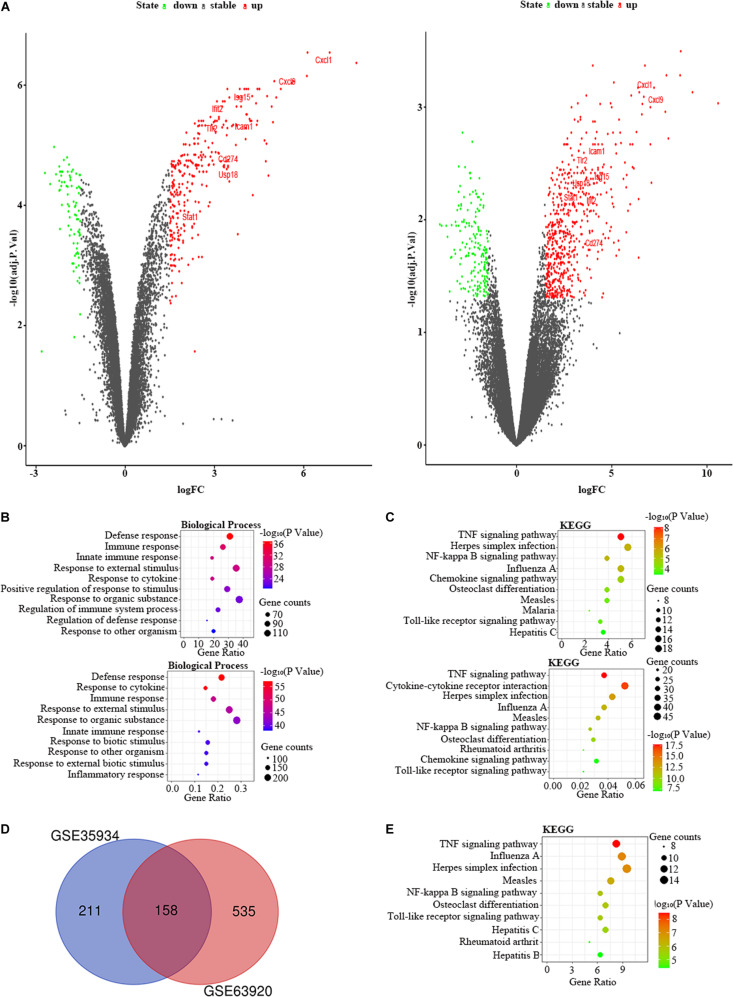
The bioinformatic analysis in heart of LPS-treated mice. **(A)** The volcano plot of DEGs in control and LPS samples in GSE35934 (left panel) and GSE63920 (right panel). FC, fold-change; green dots, downregulated genes; black dots, genes without differentially expression; red dots, upregulated genes; word in the plot, name of key genes. **(B)** The functional enrichment analysis of the biological process of DEGs in GSE35934 (upper panel) and GSE63920 (lower panel). **(C)** The functional enrichment analysis of the KEGG pathway of DEGs in GSE35934 (upper panel) and GSE63920 (lower panel). **(D)** Venn diagram illustrated the number of shared DEGs in two datasets. The intersection in red represents the shared DEGs in two datasets. **(E)** The functional enrichment analysis of the KEGG pathway of FDEGs in the heart.

We used the GSE35934 and GSE63920 datasets to detect the DEGs that were changed in the heart of the LPS group compared to the control group (Table 1). In the GSE35934 datasets, we found 369 DEGs, including 290 upregulated DEGs and 79 downregulated DEGs. In addition, in the GSE63920 datasets, we found 693 DEGs, which consist of 522 upregulated DEGs and 171 downregulated DEGs (Figure 2A). In the BP of GO analysis, both of these two datasets were mainly enriched in defense response, immune response, innate immune response, response to external stimulus, response to cytokine, response to organic substance and response to other organism (Figure 2B). In the KEGG pathway enrichment analysis, both of these two datasets were mainly enriched in the TNF signaling pathway, herpes simplex infection, NF-kappa B signaling pathway, influenza A, chemokine signaling pathway, osteoclast differentiation, measles, and Toll-like receptor signaling pathway (Figure 2C). These results therefore confirmed that these two datasets are homogeneous. We then applied the DEGs overlap analysis between the GSE35934 and GSE63920 datasets to identify the final DEGs (FDEGs) in heart. There were 158 FDEGs, including 130 upregulated FDEGs and 28 downregulated FDEGs (Figure 2D). The KEGG pathway functional enrichment analysis was showed that these FDEGs mainly enriched in TNF signaling pathway, NF-kappa B signaling pathway, and Toll-like receptor signaling pathway (Figure 2E).

**FIGURE 3:**
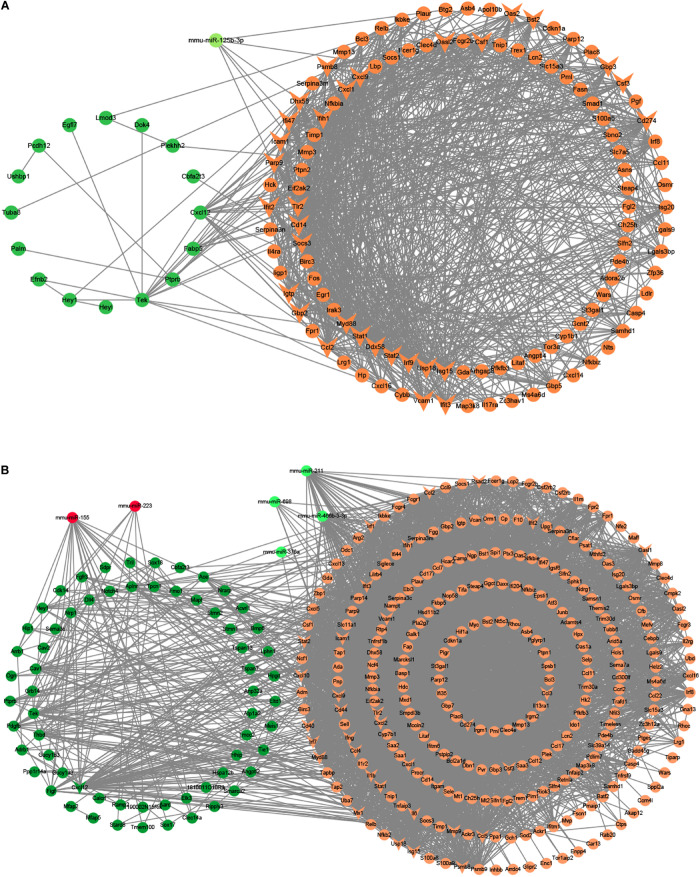
The miRNA-mRNA regulatory networks in the heart and lung of LPS-treated mice. **(A)** The miRNA-mRNA regulatory networks in the heart. **(B)** The miRNA-mRNA regulatory networks in the lung. Green nodes, downregulated genes or miRNAs; Red nodes, upregulated genes or miRNAs; V-shaped nodes, hub genes.

Besides, we used the GSE29914 to detect the DEmiRs that were changed in the heart of the LPS group compared to the control group (Table 1). The mmu-miR-125b-3p and mmu-miR-341 were downregulated in LPS-treated mice. Considering the fact that target genes are generally negative regulated by miRNAs, we intersected the predicted target genes of miRNAs with the upregulated FDEGs. We found that the FDEGs (Btg2, Ifi47, Il17ra, Pgf, St3gal1, and Myd88) were the target genes of mmu-miR-125b-3p (Table 2). Finally, we constructed the miRNA-mRNA regulatory network of the heart in LPS treated mice (Figure 3A), and we found 31 hub genes (Table 3).

**TABLE 2:** The differentially expressed miRNAs and predicted paired final differentially expressed genes in heart, lung and liver.

Organ	miRNA	adj. *P*-value	logFC	Predicted paired FDEGs
Heart	mmu-miR-125b-3p	0.02283	−1.892	Btg2, Ifi47, Il17ra, Pgf, St3gal1, and Myd88
	mmu-miR-341	0.04126	−1.693	-
Lung	mmu-miR-376a	0.003797	−1.662	Tapbp, Ccl9, and Samhd1
	mmu-miR-211	0.002431	−1.555	Fcgr4, Enpp4, Cflar, Arg2, Fcer1g, Fcgr1, Gna13, Eif2ak2, Ccl9, Sema7a, Slfn2, Tnfrsf1b, Cd40, Slc11a1, Hk2, Cxcl13, Plek, Clec4e, Pmaip1, Cd274, Asb4, Epsti1, Nfkbiz, Igsf6, Il13ra1, Oas2, Arid5a, Tor1aip2, Cp, Tnfrsf9, Socs3, Enc1, Fap, Fgl2, Ifit2, Irf1, Itgam, Cxcl9, Mmp8, Mthfd2, S100a9, and St3gal1
	mmu-miR-466b-3-3p	0.001869	−1.668	Cxcl1, Irf1, Cxcl5, Cxcl16, Riok3, Akap12, Pfkfb3, Oas3, Il1rn, Il13ra1, Slc39a14, Tor1aip2, Adamts4, Psat1, Gda, Gna13, Ifit3, Lcp2, Ccl9, Slfn4, Tnfrsf1b, Tnfrsf9, Mmp9, Sod2, Hk2, Cmpk2, Cxcl10, Rsad2, Nampt, Cd274, Ptges, Samsn1, Ms4a6d, Glipr2, Arrdc4, Itgam, Birc3, Map3k8, Fgl2, Gch1, Hdc, Ifit2, Inhbb, Junb, Mthfd2, Cxcl2, Stat1, Tnfaip2, Tnfaip3, and Enpp4
	mmu-miR-698	0.001081	−2.159	Sema7a, Usp18, Ptges, Arrdc4, Fgl2, Ifit2, Stat1, and Plek
	mmu-miR-467b*	0.004652	−1.729	-
	mmu-miR-155	0.0001072	2.077	Tmem100, 1810011O10Rik, Cxcl12, Anp32a, Hey1, Meis1, Mapt, Sox17, Elk3, Sema3c, Gucy1b3, Dll4, Tspan7, Arrb1, Atp1a2, Ace, Gucy1a3, Stmn2, Clec14a, Sdpr, Tpcn1, and Aard
	mmu-miR-223	0.00002290	2.709	Clec14a, Hip1, Arrb1, Cxcl12, 1190002N15Rik, and Thbd
Liver	mmu-miR-335-5p	0.01420	−1.767	Cd274, D10Wsu102e, Enc1, Ezr, Gbp3, Ifit3, Rnd1, Smad1, Steap4, Tmem87b, and Zc3h7a

*Adj. P-value, adjusted P-value; FDEGs, final differentially expressed genes.*

**TABLE 3:** The hub genes of heart, lung, liver and kidney.

Organ	Hub Gene
Heart	Stat1, Tlr2, Ccl2, Ifih1, Cxcl9, Myd88, Cd274, Stat2, Isg15, Ddx58, Cxcl1, Usp18, Icam1, Ifit3, Oasl2, Psmb8, Ifi47, Ifit2, Irf9, Vcam1, Csf3, Oas2, Igtp, Gbp2, Socs3, Bst2, Parp9, Cd14, Gbp3, Dhx58, and Csf1
Lung	Il6, Cxcl10, Stat1, Il1b, Tlr2, Ccl2, Ccl5, Irf7, Cxcl9, Irf1, Ifng, Itgam, Cd274, Cd40, Ccl4, Mmp9, Cxcl1, Icam1, Myd88, Cxcl2, Ifih1, Vcam1, Stat2, Isg15, Cxcl12, Psmb8, Mx1, Ccl3, Usp18, Cd44, Oasl1, Ifit3, Ifit2, and Csf3
Liver	Il6, Stat3, Stat1, Cxcl10, Il1b, Egfr, Tlr2, Ccl5, Myd88, Cd274, Cxcl9, Isg15, Icam1, Rsad2, Cd44, Ifit2, Usp18, Socs3, Ifit3, Tnfaip3, Cxcl1, Oasl2, Parp14, Dhx58, Birc3, Ifi47, Irf8, Socs1, Igtp, Cd14, Csf1, Bcl2l1, Gbp2, and Ifi44
Kidney	Stat1, Cxcl10, Irf1, Irf7, Ccl5, Usp18, Ddx58, Ifih1, Tlr2, Stat3, Oasl2, Parp14, Cd274, Isg15, Cxcl9, Rsad2, Ifit2, Jun, Irf9, Gbp2, Tlr3, Gbp3, Igtp, Psmb9, Iigp1, Rnf213, Cxcl1, Parp12, Eif2ak2, Icam1, and Gbp5

### The miRNA-mRNA Network of the Lung in LPS-Treated Mice

**FIGURE 4:**
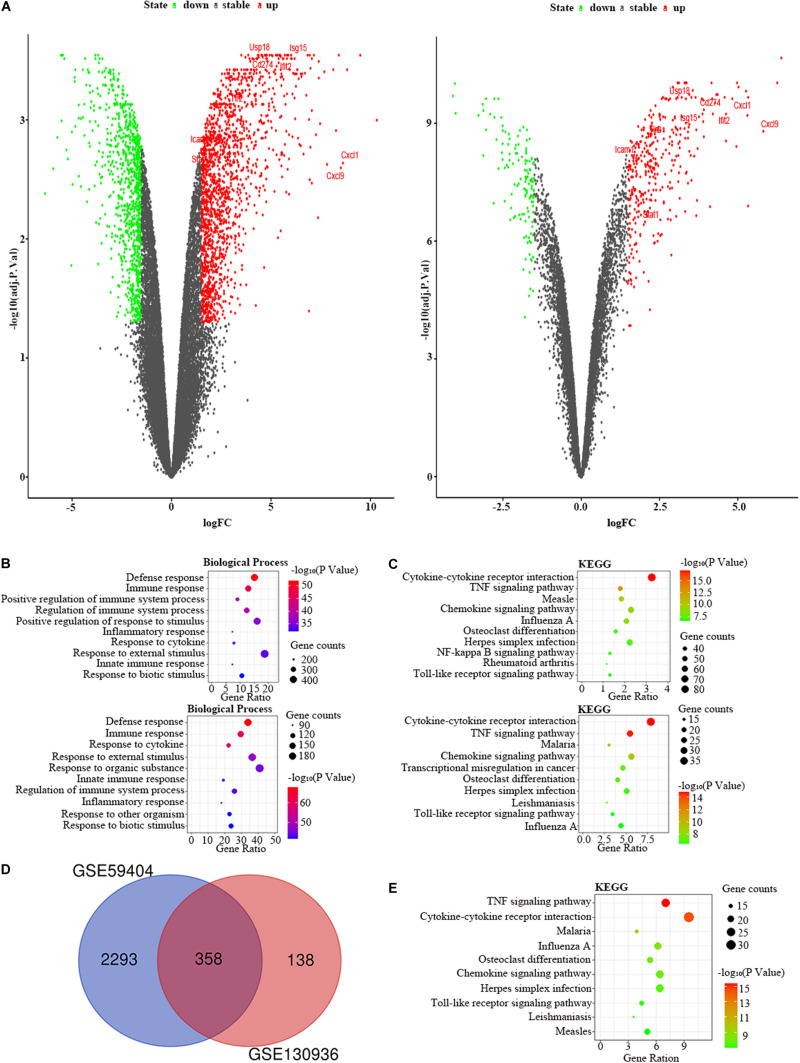
The bioinformatic analysis in the lung of LPS-treated mice. **(A)** The volcano plot of DEGs in control and LPS samples in GSE59404 (left panel) and GSE130936 (right panel). FC, fold-change; green dots, downregulated genes; black dots, genes without differentially expression; red dots, upregulated genes; word in the plot, name of key genes. **(B)** The functional enrichment analysis of the biological process of DEGs in GSE59404 (upper panel) and GSE130936 (lower panel). **(C)** The functional enrichment analysis of the KEGG pathway of DEGs in GSE59404 (upper panel) and GSE130936 (lower panel). **(D)** Venn diagram illustrated the number of shared DEGs in two datasets. The intersection in red represents the shared DEGs in two datasets. **(E)** The functional enrichment analysis of the KEGG pathway of FDEGs in the lung.

We chose the GSE59404 and GSE130936 datasets to screen the DEGs in the lung of the LPS group compared to the control group (Table 1). In the GSE59404 datasets, there were 2,651 DEGs, which consist of 1,630 upregulated DEGs and 1,021 downregulated DEGs. Moreover, in the GSE130936 datasets, there were 496 DEGs, including 362 upregulated DEGs and 134 downregulated DEGs (Figure 4A). In the BP of GO analysis, both of these two datasets were mainly enriched in defense response, immune response, regulation of immune system process, inflammatory response, response to cytokine, response to external stimulus, innate immune response and response to biotic stimulus (Figure 4B). In the KEGG pathway enrichment analysis, both of these two datasets were mainly enriched in cytokine–cytokine receptor interaction, TNF signaling pathway, chemokine signaling pathway, influenza A, osteoclast differentiation, herpes simplex infection and Toll-like receptor signaling pathway (Figure 4C). Therefore, these results indicated that these two datasets are largely homogeneous. Then we applied the DEGs overlap analysis between two datasets to confirm the FDEGs in the lung. We totally found 358 FDEGs, including 282 upregulated FDEGs and 76 downregulated FDEGs (Figure 4D). The KEGG pathway enrichment analysis was demonstrated that these FDEGs mainly enriched in TNF signaling pathway, cytokine–cytokine receptor interaction, chemokine signaling pathway and Toll-like receptor signaling pathway (Figure 4E).

Furthermore, we used the GSE36472 to screen the DEmiRs in the lung of the LPS group compared to the control group (Table 1). We found that mmu-miR-376a, mmu-miR-211, mmu-miR-466b-3-3p, mmu-miR-698, and mmu-miR-467b^∗^ were downregulated, while the mmu-miR-155 and mmu-miR-223 were upregulated in LPS-treated mice. We then intersected the predicted target genes of downregulated miRNAs with the upregulated FDEGs, and the predicted target genes of upregulated miRNAs with the downregulated FDEGs. The relationship of miRNA-paired DEGs was shown in Table 2. Finally, we constructed the miRNA-mRNA regulatory network of the lung in LPS treated mice (Figure 3B), and we found 34 hub genes (Table 3).

### The miRNA-mRNA Network of the Liver in LPS-Treated Mice

**FIGURE 5:**
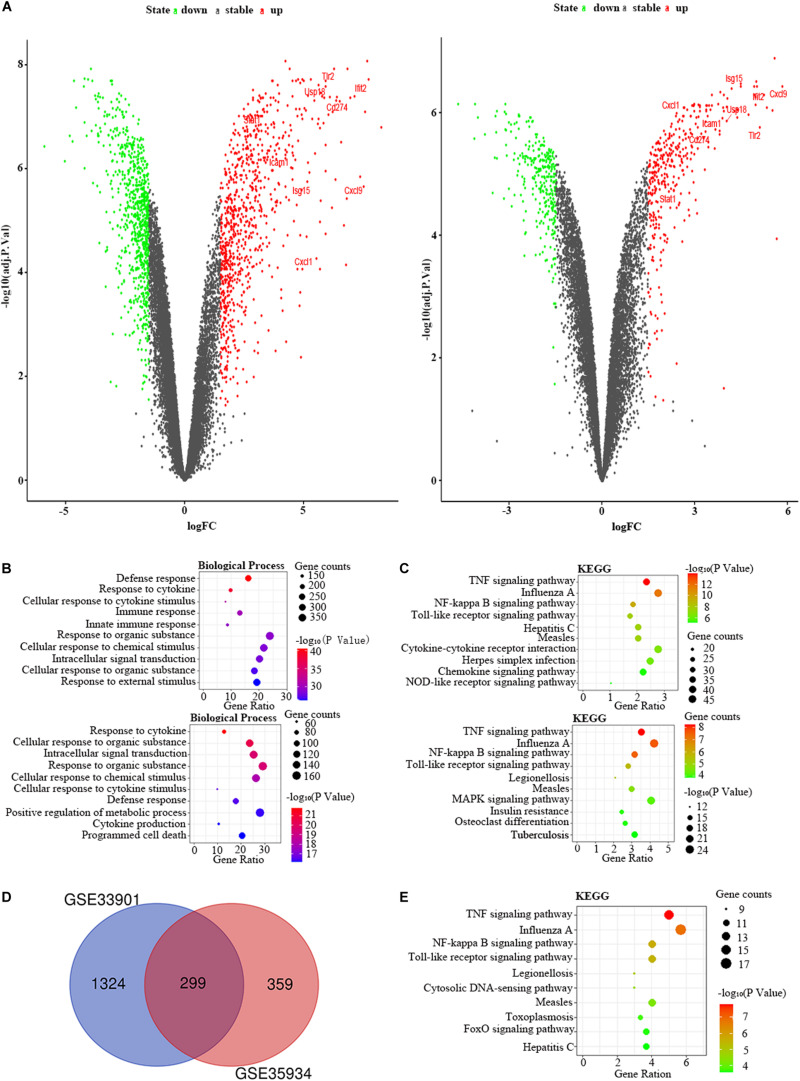
The bioinformatic analysis in the liver of LPS-treated mice. **(A)** The volcano plot of DEGs in control and LPS samples in GSE33901 (left panel) and GSE35934 (right panel). FC, fold-change; green dots, downregulated genes; black dots, genes without differentially expression; red dots, upregulated genes; word in the plot, name of key genes. **(B)** The functional enrichment analysis of the biological process of DEGs in GSE33901 (upper panel) and GSE35934 (lower panel). **(C)** The functional enrichment analysis of the KEGG pathway of DEGs in GSE33901 (upper panel) and GSE35934 (lower panel). **(D)** Venn diagram illustrated the number of shared DEGs in two datasets. The intersection in red represents the shared DEGs in two datasets. **(E)** The functional enrichment analysis of the KEGG pathway of FDEGs in the liver.

We selected the GSE33901 and GSE35934 datasets to find the DEGs that were changed in the liver of the LPS group compared to the control group (Table 1). In the GSE33901 datasets, we found 1,623 DEGs, which consist of 824 upregulated DEGs and 799 downregulated DEGs. Moreover, in the GSE35934 datasets, we found 658 DEGs, including 356 upregulated DEGs and 302 downregulated DEGs (Figure 5A). In the BP analysis, both of these two datasets were mainly enriched in defense response, response to cytokine, cellular response to cytokine stimulus, response to organic substance, cellular response to chemical stimulus, intracellular signal transduction, and cellular response to organic substance (Figure 5B). In the KEGG pathway enrichment analysis, both of these two datasets were mainly enriched in TNF signaling pathway, influenza A, NF-kappa B signaling pathway, Toll-like receptor signaling pathway, measles (Figure 5C). These results therefore indicated that these two datasets are similar. Then we applied the DEGs overlap analysis between these two datasets to identify the FDEGs in the liver. We identified 299 FDEGs, including 202 upregulated FDEGs and 97 downregulated FDEGs (Figure 5D). The KEGG pathway functional enrichment analysis was showed that these FDEGs mainly enriched in TNF signaling pathway, NF-kappa B signaling pathway, Toll-like receptor signaling pathway, cytosolic DNA-sensing pathway and FoxO signaling pathway (Figure 5E).

**FIGURE 6:**
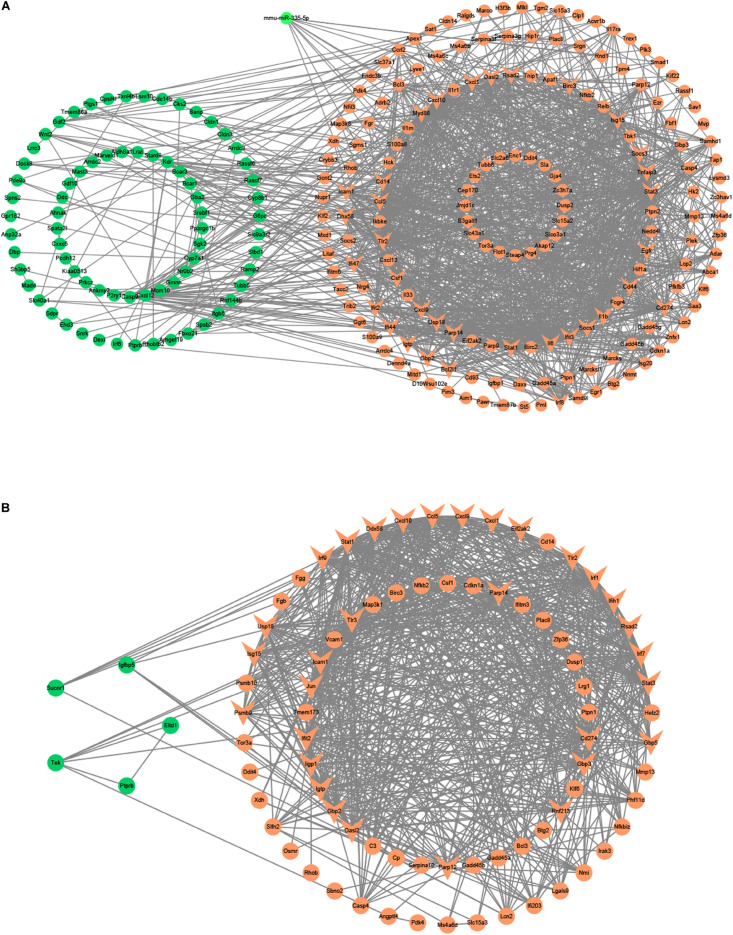
The miRNA-mRNA or PPI regulatory networks in the liver and kidney of LPS-treated mice. **(A)** The miRNA-mRNA regulatory networks in the liver. **(B)** The PPI regulatory networks in the kidney. Green nodes, downregulated genes or miRNAs; Red nodes, upregulated genes or miRNAs; V-shaped nodes, hub genes.

Besides, we used the GSE33902 to find the DEmiRs in the liver of the LPS group compared to the control group (Table 1). We found that mmu-miR-335-5p was downregulated in LPS-treated mice. Furthermore, we intersected the predicted target genes of downregulated miRNAs with the upregulated FDEGs. We found that the FDEGs (Cd274, D10Wsu102e, Enc1, Ezr, Gbp3, Ifit3, Rnd1, Smad1, Steap4, Tmem87b, and Zc3h7a) were the target genes of mmu-miR-335-5p (Table 2). Finally, we constructed the miRNA-mRNA regulatory network of the liver in LPS treated mice (Figure 6A), and we found 34 hub genes (Table 3).

### The PPI Network of Kidney in LPS-Treated Mice

**FIGURE 7:**
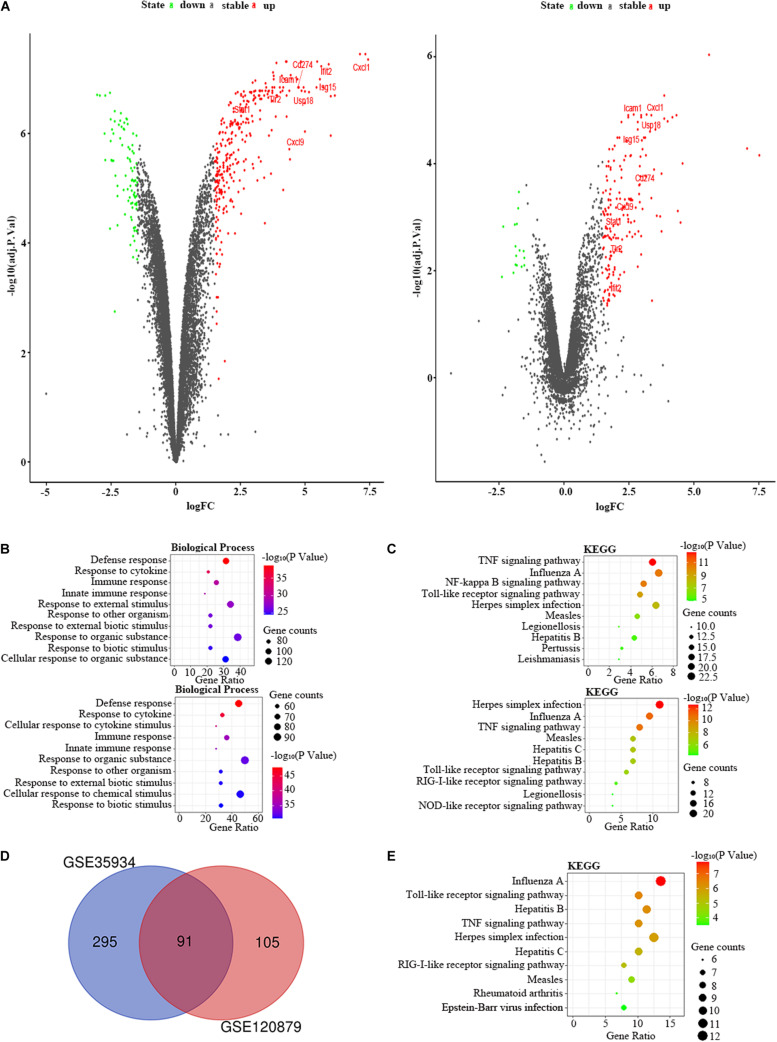
The bioinformatic analysis in the kidney of LPS-treated mice. **(A)** The volcano plot of DEGs in control and LPS samples in GSE35934 (left panel) and GSE120879 (right panel). FC, fold-change; green dots, downregulated genes; black dots, genes without differentially expression; red dots, upregulated genes; word in the plot, name of key genes. **(B)** The functional enrichment analysis of the biological process of DEGs in GSE35934 (upper panel) and GSE120879 (lower panel). **(C)** The functional enrichment analysis of the KEGG pathway of DEGs in GSE35934 (upper panel) and GSE120879 (lower panel). **(D)** Venn diagram illustrated the number of shared DEGs in two datasets. The intersection in red represents the shared DEGs in two datasets. **(E)** The functional enrichment analysis of the KEGG pathway of FDEGs in the kidney.

We applied the GSE35934 and GSE120879 to explore the DEGs in the kidney of the LPS group compared to the control group (Table 1). In the GSE35934 datasets, there were 386 DEGs, which consist of 298 upregulated DEGs and 88 downregulated DEGs. Moreover, in the GSE120879 datasets, there were 196 DEGs, including 180 upregulated DEGs and 16 downregulated DEGs (Figure 7A). In the BP analysis, both of these two datasets were mainly enriched in defense response, response to cytokine, immune response, innate immune response, response to other organism, response to external biotic stimulus, response to organic substance and response to biotic stimulus (Figure 7B). In the KEGG pathway enrichment analysis, both of these two datasets were mainly enriched in TNF signaling pathway, influenza A, Toll-like receptor signaling pathway, herpes simplex infection, measles, legionellosis, and hepatitis B (Figure 7C). Therefore, these results confirmed that these two datasets are largely similar. Then we applied the DEGs overlap analysis between these two datasets to identify the FDEGs in the kidney. There were 91 FDEGs, including 85 upregulated FDEGs and 6 downregulated FDEGs (Figure 7D). The KEGG pathway functional enrichment analysis was showed that these FDEGs mainly enriched in TNF signaling pathway, Toll-like receptor signaling pathway and RIG-I-like receptor signaling pathway (Figure 7E). Finally, we constructed the PPI regulatory network of the kidney in LPS treated mice (Figure 6B), and we identified 31 hub genes (Table 3).

### Identifying Key Genes and Drugs in Four Organs

**FIGURE 8:**
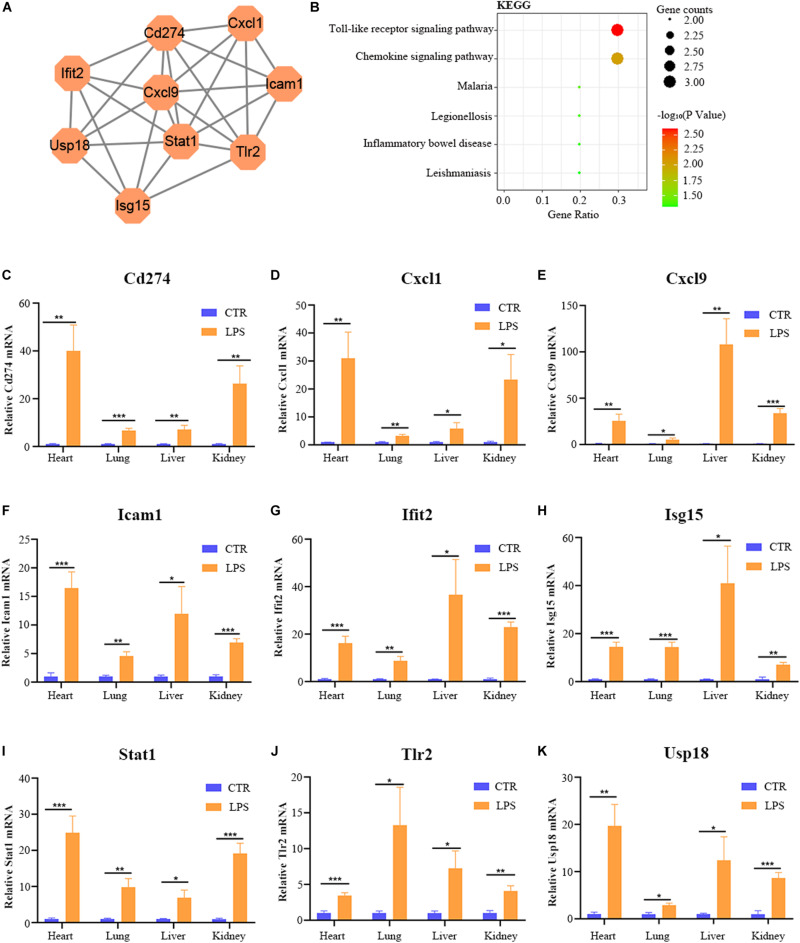
The bioinformatic analysis and verification of key genes. **(A)** The PPI regulatory networks of key genes. **(B)** The functional enrichment analysis of the KEGG pathway of key genes. **(C–K)** The relative mRNA expression level of nine key genes (Cd274, Cxcl1, Cxcl9, Icam1, Ifit2, Isg15, Stat1, Tlr2, and Usp18) in four organs (heart, lung, liver, and kidney) of LPS-treated mice by RT-qPCR. Data are presented as the mean ± SEM (*n* = 6). CTR, control group; LPS, lipopolysaccharide group. *P < 0.05 versus control group, **P < 0.01 versus control group, and ***P < 0.001 versus control group.

By the intersection of the hub genes of four organs, we found the nine key genes in LPS induced multi-organ damage mice, including Cd274, Cxcl1, Cxcl9, Icam1, Ifit2, Isg15, Stat1, Tlr2, and Usp18. The PPI network of these key genes was shown in Figure 8A. KEGG enrichment analysis showed that these key genes mainly enriched in Toll-like receptor signaling pathway and chemokine signaling pathway (Figure 8B). Then we constructed the LPS-induced mice model, and we used the RT-qPCR methods to verify that the nine key genes (Cd274, Cxcl1, Cxcl9, Icam1, Ifit2, Isg15, Stat1, Tlr2, and Usp18) are increased in different organs (heart, lung, liver, and kidney) (Figures 8C–K). Moreover, we predicted the drugs for these genes by DGIdb software. The Cd274 may be targeted by ATEZOLIZUMAB, AVELUMAB, NIVOLUMAB, and PEMBROLIZUMAB. Besides, the Icam1 may be targeted by NATALIZUMAB and LIFITEGRAST. Furthermore, the Isg15 may be targeted by IRINOTECAN.

## Discussion

Endotoxin-induced multi-organ failure is common in ICU patients and related to the increased risk of death (Hurley et al., 2012; Huang et al., 2016; Li et al., 2019). LPS is an indispensable content for the pathogenicity of endotoxemia, and the LPS-induced mice model is a frequently used animal model for endotoxemia (Pils et al., 2010). Mapping the miRNA-mRNA network and digging the common mechanism of multi-organ may provide new treatment targets. So far, there was no study on the multi-organ miRNA-miRNA regulatory network analysis in LPS-treated mice. In our study, we downloaded the GEO datasets from the NCBI website and analyzed the datasets by bioinformatic methods. First, we identified FDEGs and DEmiRs in the heart (158 FDEGs and 1 DEmiRs), lung (358 FDEGs and 7 DEmiRs), liver (299 FDGs and 1 DEmiRs), and kidney (91 FDEGs) through differential gene or miRNA analysis. Subsequently, we used these FDEGs to perform pathway enrichment analysis, and we found that TNF signaling pathway and Toll-like receptor signaling pathway were enriched in FDEGs of all these organs. We then constructed the miRNA–mRNA network to screen the hub genes in the heart (31 hub genes), lung (34 hub genes), liver (34 hub genes), and kidney (31 hub genes). Finally, we identified and verified 9 key genes (Cd274, Cxcl1, Cxcl9, Icam1, Ifit2, Isg15, Stat1, Tlr2, and Usp18), and we predicted seven potential drugs for multi-organ damage in LPS-induced endotoxemia.

Previous studies have shown that LPS promotes the activation of downstream inflammatory signaling pathways by activating TLR4 related pathways (Ryu et al., 2017). Subsequently, over-activated proinflammatory factors lead to the target-organ damage (Cochet and Peri, 2017). Similarly, our study confirmed that FDEGs of heart, lung, liver, and kidney were enriched in a Toll-like receptor signaling pathway and TNF signaling pathway, which produced inflammatory factors. In addition, our study also found that multi-organ’s FDEGs are also enriched in some viral infection related signaling pathways, such as influenza A, measles, and herpes simplex infection pathways (Figures 2E, 4E, 5E, 7E). These results indicated that LPS may be similar to some viruses in the pattern of immune system activation. These results may thus provide a new direction for future research in this field.

microRNA plays an important role in many pathophysiological processes, such as growth and development, inflammation, and tumor (Hammond, 2015). Recently, there have been clinical trials using miRNAs as a treatment method for diseases (Beg et al., 2017). Previous studies have found that the expression level of miRNAs has changed in different organs of LPS-treated mice (Hsieh et al., 2012). Interestingly, our study also found DEmiRNAs in the heart, lung, and liver (Table 2). By predicting the target genes of DEmiRs and constructing the miRNA-mRNA regulatory network, we found that most miRNAs may function by regulating the expression of FDEGs. In the future, these altered miRNAs may be a new therapeutic target for endotoxin-mediated multi-organ damage.

In our research, we identified nine key genes in LPS-mediated multi-organ damage. PPI network confirmed that these nine genes (Cd274, Cxcl1, Cxcl9, Icam1, Ifit2, Isg15, Stat1, Tlr2, and Usp18) were interacting with each other (Figure 8A). KEGG pathway enrichment analysis showed that these genes enriched in the Toll-like receptor signaling pathway and chemokine signaling pathway, which further confirmed their significance. CD274, referred to as PDL1, functions as a T-cell immune inhibitor by binding to its receptor PD1. In endotoxemia, NK-κB and C5a induced Cd274 expression to promote immune suppression, which may indirectly increase the uncontrolled inflammation (Huang et al., 2013; An et al., 2016). CXCL9 and CXCL11 are important chemokines to promote inflammatory cells transfer to the target organ. In LPS-mediated acute lung injuries, ROS enhanced the pulmonary epithelial cells to secrete CXCL9, which increased B-cell transfer to bronchoalveolar fluid (Zhang et al., 2018). Besides, other studies showed that LPS can induce the expression of CXCL11, an IFN-inducible T-cell alpha chemoattractant, in macrophages (Khabbazi et al., 2016; Ye et al., 2018). Icam1, encoding a cell surface glycoprotein, participated in leukocyte adhesion and antigen processing or presentation of immune cells to promote system inflammation or immune response. Previous studies confirmed that ICAM-1 was related to the development of multi-organ failure, and the persistent increase of ICAM-1 in plasma of sepsis-induced multi-organ failure may indicate a proinflammatory endothelium phenotype (Whalen et al., 2000; Amalakuhan et al., 2016). Ifit2, a type-I interferon-induced protein, aggravated the outcome of LPS induced endotoxin shock mice by increasing the expression of proinflammatory factors, such as TNF and IL-6 (Siegfried et al., 2013). Isg15, an IFN-induced ubiquitin-like protein, upregulated and largely binds to target protein during bacterial infection to function via the JAK-STAT pathway (Kim and Zhang, 2003). Stat1, a transcription activator, can promote the LPS-mediated cytokine production (IL-6 and IL-12 p40) via non-canonical phosphorylation (Metwally et al., 2020). Moreover, another study showed that the JAK2/STAT1 pathway can promote HMGB1 translocation to enhance inflammation in the LPS-induced lung injury model (Liu et al., 2019). Tlr2, a member of the Toll-like family, acts as a vital role in innate immunity. In addition, Wang et al. found that HMGB1 could induce M1 macrophages polarization via TLR2 pathways in LPS-mediated lung injury (Wang et al., 2020). Usp18, a ubiquitin-specific protease, cleaves only ISG15 conjugates. Furthermore, MacParland et al. (2016) demonstrated that USP18 upregulated and blocked IFN stimulated gene expression to affect the innate immunity in LPS-induced hepatocytes. Our study indicated that these key genes may be critical, and we predicted some potential drugs in endotoxin-induced multi-organ failure. Most of the existing studies, however, only focus on the influence of a single organ in endotoxemia. We therefore need more experimental studies on multiple-organ failure induced by endotoxemia in the future.

In this study, we have identified key genes and pathways in endotoxin-induced multiple organ damage. We verified the expression level of these key genes. However, because most of the data used are from public datasets, there are still some limitations to our research. First of all, although we want to use a variety of different programs to predict target genes of miRNAs to increase the reliability of the conclusion, there is still the possibility of false positive. We will perform *in vivo* and *in vitro* experimental verification to confirm the miRNA-mRNA regulatory network in the future. Secondly, the mouse sample datasets we selected were from different strains, ages, and genders, and we have therefore taken measures to compare the homogeneity of different databases. Before screening the hub gene of a certain organ, we performed and compared the gene function and pathway enrichment analysis in every two datasets. The results of enrichment analysis showed these databases are enriched in almost the same gene function terms and pathways, suggesting that these databases are homogeneous. By taking the intersection of the analysis results of different datasets, we finally retain the genes and pathway changes related to LPS treatment, which can increase the scope of application of our study. Our results indicated that there are significant changes in immune and inflammatory pathways in LPS-mediated endotoxemic mice of different backgrounds. Third, in the study of the kidney, we did not find miRNA microarray data. Constructing a miRNA-mRNA network in the kidney may be helpful to further improve our research. We will therefore further research the role and mechanism of these key genes in endotoxemia-induced multi-organ failure patients or animal models in the future.

## Conclusion

In conclusion, we describe a multi-organ miRNA-mRNA regulatory network in LPS treated mice to elucidate the underlying molecular pathways and key genes. Our results indicated that the Toll-like receptor signaling pathway and the TNF signaling pathway is the main pathway in endotoxin-induced multi-organ damage. Besides, we identified nine key genes (Cd274, Cxcl1, Cxcl9, Icam1, Ifit2, Isg15, Stat1, Tlr2, and Usp18) and potential drugs for endotoxin-induced multi-organ failure. Our data provide a new sight and potential target for future therapy in endotoxin-induced multi-organ failure.

## Data Availability Statement

The datasets presented in this study can be found in online repositories. The names of the repository/repositories and accession number(s) can be found below: https://www.ncbi.nlm.nih.gov/, GSE35934; https://www.ncbi.nlm.nih.gov/, GSE63920; https://www.ncbi.nlm.nih.gov/, GSE29914; https://www.ncbi.nlm.nih.gov/, GSE59404; https://www.ncbi.nlm.nih.gov/, GSE130936; https://www.ncbi.nlm.nih.gov/, GSE36472; https://www.ncbi.nlm.nih.gov/, GSE33901; https://www.ncbi.nlm.nih.gov/, GSE33902; https://www.ncbi.nlm.nih.gov/, GSE120879.

## Ethics Statement

The animal study was reviewed and approved by Institutional Animal Care and Use Committee of Sun Yat-sen University, Guangzhou, China.

## Author Contributions

ZY, CZ, and YL conceptualized the study. CZ and LZ performed the animal study. CZ, ZP, and YL performed the writing and draft preparation of the manuscript. CZ and ZY wrote, reviewed, and edited the manuscript. CZ, ZL, and LZ visualized the study. ZY and JL supervised the study. All authors contributed to the article and approved the submitted version.

## Conflict of Interest

The authors declare that the research was conducted in the absence of any commercial or financial relationships that could be construed as a potential conflict of interest.

## Footnotes

1 http://www.ncbi.nlm.nih.gov/geo/

2 https://david.ncifcrf.gov

3 http://c1.accurascience.com/miRecords/

4 www.dgidb.org
